# Is there an optimal place to hold the endotracheal tube during direct laryngoscopy for patients undergoing surgery under general anesthesia? Protocol for a randomized controlled trial

**DOI:** 10.1186/s13063-021-05635-5

**Published:** 2021-10-09

**Authors:** Manisha Sahoo, Swagata Tripathy, Nitasha Mishra

**Affiliations:** AIIMS Bhubaneswar: All India Institute of Medical Sciences, Bbsr, Bhubaneswar, Odisha India

**Keywords:** Direct laryngoscopy, Intubation difficulty score, Endotracheal intubation, Site of holding endotracheal tube, Torque, Time to intubation

## Abstract

**Background:**

Endotracheal intubation by direct laryngoscopy is a widely performed lifesaving technique. Although there are guidelines for optimal size and depth of insertion of an endotracheal tube (ETT) for successful intubation, there is no consensus on the point at which it should be held along its length. This will arguably affect the time, ease, and success of the technique due to a difference in visualization and torque applied to the ETT after glottic visualization. We aim to compare the effect of 2 different sites of holding the ETT on time to intubation (TTI), intubation difficulty scale (IDS), and complications.

**Methods:**

ASA 1–2 patients (>18 years) posted for surgery under general anesthesia, undergoing supervised intubation by anesthesia trainees (experience < 18 months), will be included. Patients with an anticipated difficult airway or unanticipated difficulty—CL grade 3 or 4 requiring the use of airway adjuncts—will be excluded. Patients will be randomized by a computer-generated number list, and allocation concealed with opaque sealed envelopes. The two sites for holding the ETT will be group 1 at 19 cm and group 2 at 24 cm. ETT marked at the selected site will be handed by the technician once the optimum position of the table, patient, and laryngoscopic view is confirmed by the intubator. The entire procedure will be video recorded. Two blinded assessors will independently review the videos to document the time to intubation and intubation difficulty score. A postoperative sore throat will be recorded.

**Sample size:**

To detect a 20% difference in time to intubation between groups with a significance level of 5% and power of 85%, we will need a total of 298 patients. Accounting for data loss, we plan to recruit 180 patients in each group.

**Discussion:**

This will be the first study to assess whether the site of holding the tube has any impact on the ease and time taken for intubation. The findings of this study will provide scientific evidence for suggesting an appropriate place for holding the ETT during direct laryngoscopy procedures.

**Trial registration:**

Clinical Trials Registry India CTRI/2019/09/021201

## Introduction

### Background and rationale {6a}

Laryngoscopic endotracheal intubation (LEI) is a fundamental procedure for securing the airway, performed for resuscitation in emergency scenarios or elective conditions such as general anesthesia. Successfully placing the endotracheal tube (ET) in the trachea is a skill learned over time. Successful intubation by anesthesiology trainees has been defined by Konrad et al. [1] as “adequate technical performance” without any staff assistance. Traditional teaching guides trainees from available evidence on the optimal position of patients’ head and neck [2], the size and depth of the endotracheal tube [3, 4], type of laryngoscope blade [5], method of laryngoscopy, and direction of bevel for intubation success [6].

**Fig. 1:**
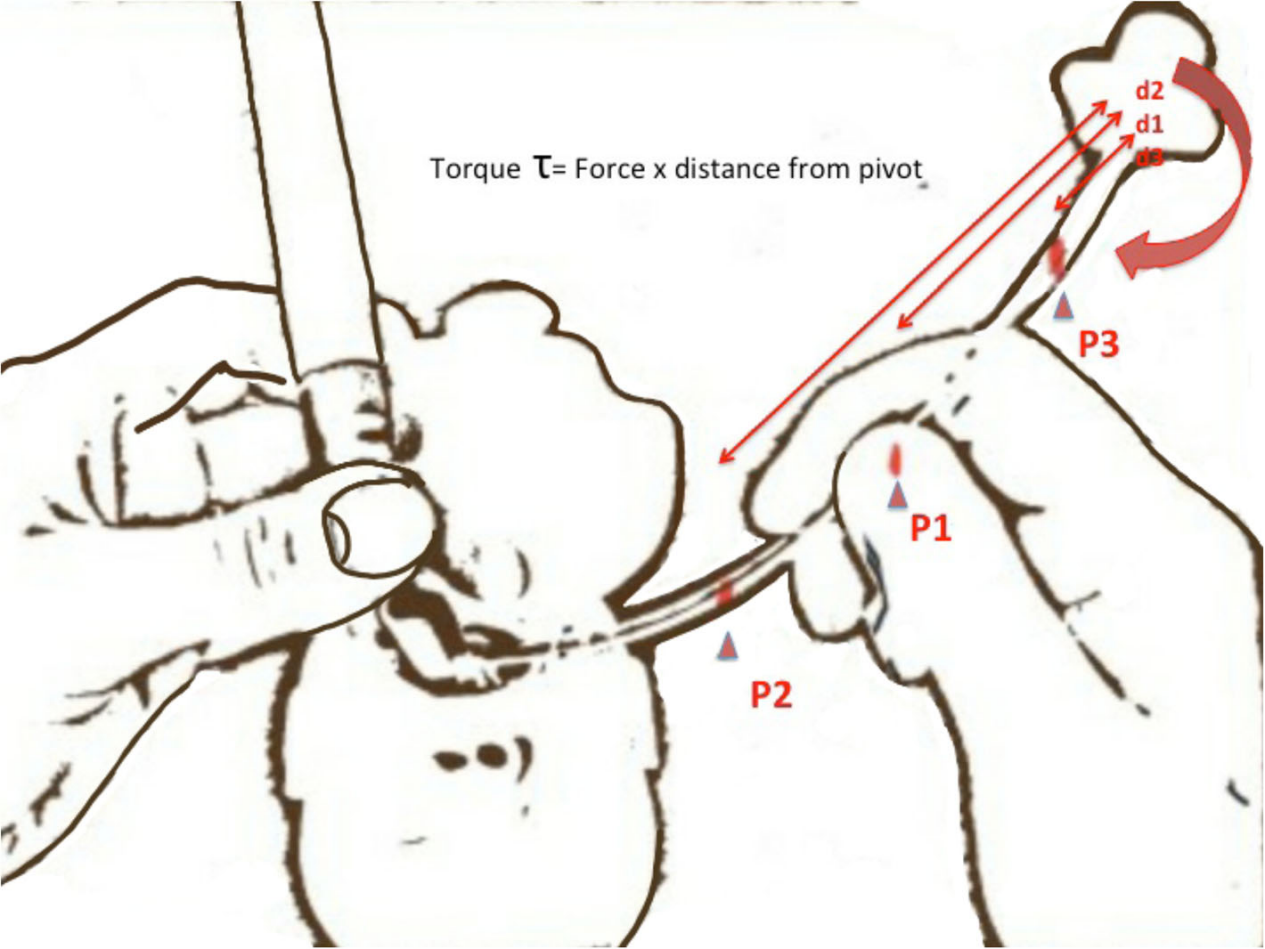
Figure illustrating how the torque varies with the point at which the endotracheal tube is held ( P1, 2, 3); as thedistance from the tip increases ( P3), lesser is the torque making for easier fine adjustments to the movements ofthe tube tip
- With inexperienced trainees, juggling between maintaining the forces and torque on the laryngoscope with the left hand and coordinating the right-handed movements might make the process more difficult [7]

This study’s senior investigator has more than 20 years of experience as a teacher of graduate and post-graduate trainees (medical and paramedical) in emergency medicine, anesthesia, and critical care. Over years of witnessing thousands of laryngoscopic intubations, she observed that more experienced personnel hold the ET farther away from the patient-end than do trainees. Discussions among peers confirmed that others shared her opinion. The consensus was that by learning from the experience of multiple intubations, experienced personnel realized (subconsciously) that the further the tube is held from the tip,

- The better is the resultant glottic view—the right hand gripping the tube has a lesser chance of coming in the field of vision during the wrist movements
- The easier it is to fine-adjust the movement of the tip of the ET close to the glottis. As the pivot (point of rotation of the tube, usually the thumb) moves away from the tip, the negative torque exerted by the distal part of the ET decreases, thereby making it easier to manipulate and guide it into the glottic opening (Fig. 1)

A literature search resulted in only one mannikin-based study: it hypothesized that the position of holding the tube would logically affect the path of vision and torque applied to the ET during the procedure. The farther the tube is held, with the elbow close to the chest, the greater the positive torque will be generated when the cuff passes the incisors, affecting the ET tip’s tilting angle [3]. There is no data available in human studies. Experts hold the ET at an optimal position by virtue of their experience. However, without evidence and guidelines for the optimal position, trainees tend to hold it over a range of places.

We aim to compare the time for intubation, complications, and the success of LEI performed by trainees, between two groups: one holding the ETT at 19cm from the tip and the other at 24 cm .

### Objectives {7}

#### Aims

To find if the place where the ET is held (site) affects LEI after a good laryngoscopic view is attained.

#### Objectives

##### Primary objective

To compare the time to intubation (TTI) between two groups of trainees holding the ET at two different (predetermined) sites.

##### Secondary objectives

To compare between the groups

● Rate of success of first-pass intubation

● Intubation difficulty score (IDS)

● The subjective ease of intubation, as assessed by the trainee, the supervising anesthetist, and a blinded assessor

● Airway-related complications

### Trial design {8}

This will be a randomized, controlled, surgeon-, patient-, and assessor-blinded single-center equivalence trial with two parallel groups and a primary endpoint of time to intubation during direct laryngoscopy for general anesthesia. Randomization will be performed as simple randomization with 1:1 allocation.

## Methods: participants, interventions, and outcomes

### Study setting {9}

This study will be conducted at the Department of Anesthesiology and Critical Care at All India Institute of Medical Sciences, Bhubaneswar, Odisha, India. AIIMS Bhubaneswar is a 900-bed tertiary care academic hospital, a designated center of national importance. It runs graduate and post-graduate training courses for a large number of medical and surgical specializations. The study is planned in the main operation theater complex with 25 operation theaters spread over four floors. Annually, more than 12,000 surgeries take place under general anesthesia.

### Eligibility criteria {10}

The participants included in the study will be:

i. Patient: Adult patients (age > 18 years) with ASA I to II functional status undergoing elective/emergency surgical procedures under general anesthesia who can understand and provide valid consent for the procedure. The exclusion criteria will be patients less than 18 years of age, American Society of Anesthesiologists (ASA) > 2 functional status, patients having Cormack Lehane (CL) grading more than 2 on direct laryngoscopy, use of airway adjuvants, and refusal to give consent for the study.
ii. Intubator: Intervention (intubation) will be performed by trainee anesthesia residents who have less than 18 months of experience. As a part of the curriculum, training in the theory of airway management and mannikin intubations will ensure that all the trainees have experience of a minimum of 40 intubations on mannikins or patients. A consultant anesthetist will supervise all intubations per protocol.
iii. Procedure: All procedures will be video recorded from the time patient is positioned after induction, and the laryngoscope is asked for, to after confirmation of correct tube position (this may be visual, auscultatory, or EtCO2 guided). Procedures where the trainee deviates from the methodology, improper table position, CL grade found > 2, use of a bougie, stellate, or other airway adjuncts, or take-over by the consultant (unanticipated difficult airway), will be video recorded but excluded from the analysis. This will reduce confounding the time to intubation due to poor visualization of the laryngeal inlet.

A few cases will be tried with a Go-Pro camera, hoping to ascertain data for the exact laryngoscopy view (with the understanding that the visual axis of the camera will not be identical to the LEI, and the forehead mounted camera might make the process more cumbersome for the trainee).

#### Dropout criteria

Any change in grip after holding the ETT, giving backward-upward-right-posterior pressure (BURP) to the larynx after holding ETT, or manipulation of the ETT like changing the curvature of the tube or rotating it by 180° will be video recorded but will be excluded from the final analysis. If any video has a conflict of opinion between the assessors regarding time to intubation or method of intubation, it will be excluded from the final analysis.

#### Who will take informed consent? {26a}

The investigators will identify eligible participants based upon inclusion and exclusion criteria. A detailed written and oral information on the study will be provided to the patients in an understandable language (English/Odia), and the investigators will take written informed consent.

#### Additional consent provisions for collection and use of participant data and biological specimens {26b}

The written consent will include information regarding videography of the procedure of intubation and the use of relevant data for academic purposes.

### Interventions

#### Explanation for the choice of comparators {6b}

##### An explanation for the selection of the intubator

It was hypothesized that we would need to include trainees who had a good knowledge of and experience for proper laryngoscopy, so that poor vision of the glottis would not confound the time to intubation. We did not want to include personnel who were very experienced as various other subtle “learned/subconscious” maneuvers would be difficult to predict or control for. As per previous studies, 40–60 LEIs on patients or manikins were considered adequate [1, 4].

##### An explanation for the site of holding the ET

The two sites of holding the ET were selected, based on a pilot study involving video recording the procedure of intubation by 54 experienced (>3 years) anesthesiologists. The mean site of holding the tube was 21.3 cm with a standard deviation (SD) of 2.5 cm. Hence, two groups’ extremes from the mean ± 1 SD were chosen and rounded up to the nearest number, one at 19 cm (group 1) and the other at 24 cm (group 2).

#### Intervention description {11a}

##### Equipment

All emergency airway and resuscitation equipment will be available. ETCO2 enabled anesthesia machines, and SpO2 probes will be in place.

##### Personnel

1. The consultant anesthetist and trainee will position the patient and prepare for intubation.

2. The operation theater technician (not a part of the research team) will select a sealed envelope after the patient enters the theater and the WHO checklist is completed. He will then mark the endotracheal tube at the site the patient has been randomized to19 or 24 cm with a transparent sticker.

3. A trainee technician (not a part of the research team) will be positioned in a way such that the entire process is visible and will video record the procedure.

##### Position

The head position will be optimized to get the best possible view of the vocal cords. The “sniffing position” that aligns the oral, pharyngeal, and laryngeal axes will be achieved by elevating the patient’s head, extending the head at the neck, and aligning the ears horizontally with the sternal notch. In morbidly obese patients, rolls will be utilized [2]. The operating table’s position will be at the height of the intubator’s xiphoid process and her/his eyes about 1 foot (∼30 cm) above the patient’s face to provide proper angles and distances for laryngoscopy [5]. If the table position is not “exact” (in the videography), but the intubator has declared a CL grade 1 or 2 view, the procedure will be considered adequate as the head and neck position has been considered less critical for a successful LEI [4].

##### Laryngoscope

All procedures will be performed with a curved blade Macintosh laryngoscope, size ¾ as appropriate for the patient.

##### Endotracheal tube

All patients will be intubated with new, single-use, room temperature, PVC, high-volume, low-pressure, cuffed ET of appropriate size. All tubes are procured from the same company at the institute. The use of a stylet or bougie will not be allowed in the first attempt.

##### Medications

All the patients in both groups will undergo a standard anesthesia technique using Fentanyl 2 mcg per kg for analgesia, 2.5 mg per kg of propofol for induction, and 0.07–0.1 mg/kg of vecuronium for muscle relaxation. Maintenance of anesthesia will be with isoflurane or sevoflurane, aiming for an agent-appropriate MAC intubation.

#### Technique

After induction and a minimum of 3 min of administering the paralytic agent, the intubator will perform the laryngoscopy. Once the intubator declares that the CL grade is optimal (CL < 3), it will be confirmed by the faculty or senior either verbally or visually. Once declared as an “optimum laryngoscopic view,” the tube will be handed over by the technician to the intubator and prior instruction will be given to take the first attempt at the marked point, with the elbow close to the body, with the thumb at the plastic marker, index, and middle fingers opposite [2]. If the above will be difficult, then any change in methodology will be allowed as per the faculty’s decision and will be documented from the video. The whole procedure will be videographed.

#### Post-procedure

Post-extubation, the airway will be checked for any complications like bleeding or sore throat in the immediate postoperative period. If there is an event of failed intubation by the trainee, then the senior will be assessed during the process of intubation.

### Criteria for discontinuing or modifying allocated interventions {11b}

If the intubator cannot intubate at the given tube mark, he will be allowed to change the site of grip—the site change will be noted from the video. If he/she still fails, the consultant can take over.

### Strategies to improve adherence to interventions {11c}

All residents and faculty will be informed about the study protocol for improving adherence. The intubator will be instructed about the procedure, and a transparent film shall be marked on the tube to guide the intubator on the site of holding.

### Relevant concomitant care permitted or prohibited during the trial {11d}

For the study, no standard treatment shall be withheld. No differential treatment or partiality in any form will be extended to a patient who has refused to be a part of the study or who voluntarily withdraws from the study at any point.

### Provisions for post-trial care {30}

In the unlikely event of any untoward events arising from the intervention, the researchers will treat them within the available institutional facilities’ bounds.

The center where the study is done is a 900-bedded institute. It is well equipped with adequate resources and facilities to cater to any possible untoward events arising because of the intervention.

### Outcomes {12}

Primary outcome measures: time to intubation (TTI) in both groups. The TTI will be calculated from holding the ET to removing the laryngoscope—after declaring a tube passed “under vision.” The frame has been chosen to reduce bias due to laryngoscopy problems or delay due to attaching the circuits and ventilating till capnographic confirmation of correct tube position. The primary outcome will be described in seconds.

Secondary outcome measures assessed will be first-pass intubation success rate; intubation difficulty scores (IDS) [6]; ease of the intubation assessed by the intubator, faculty, and a blinded assessor unrelated to the study on the Likert scale (0—very easy to 5—extremely difficult); and incidence of complications (bleeding or sore throat) in each group. The IDS comprises of attempt (in no), additional operators (if any, then in no), alternative technique: stylet/bougie, Cormack Lehane grading (CL grade: I/II/III/IV), lifting force: normal (0)/increased (1), external laryngeal pressure required during insertion of the tube: no (0)/yes (1), and position of vocal cords: abducted (0)/adducted (1).

Assessments from the video footage: These assessments will be done by two different anesthesiologists independently. The mean values will be used for analysis after disputes/disagreements (if any) are settled by a third person not belonging to the research team. This will reduce bias due to possible errors in time calculations.

### Participant timeline {13}

**Table:** 

	Study period				
	Enrolment allocation	Post-allocation			
Timelines	Before surgery	Before intubation (induction to mask ventilation)	Intubation	Post-intubation	Post-extubation
**Enrollment**					
Informed consent	X				
Eligibility criteria	X				
Allocation	X				
**Interventions**					
Group 1 (19 cm)					
Group 2 (24 cm)					
**Assessments**					
Demographic characteristics	**X**				
Airway assessment	**X**	X			
Experience of the intubator (in months)		X			
Time of holding of ET (T1)			**X**		
Time of removal of laryngoscope (T2)				**X**	
Time to intubation (from T1 to T2)			**X**	**X**	
Intubation difficulty score			**X**		
Any change in the site of holding the tube			**X**		
Videography during intubation		**X**	**X**	**X**	
Ease of intubation on Likert scale by the intubator			**X**	**X**	
Ease of intubation assessed by faculty			**X**	**X**	
Ease of intubation assessed by a blinded assessor					**X**
Incidence of airway trauma (bleeding/sore throat )			**X**		**X**

### Sample size {14}

Based on the pilot study, the mean time to intubation was found to be 8.83 s, a standard deviation of 4.07 s, and a variance of 16.57 s. Using this information—an allocation ratio of 1:1, to ascertain a 20% difference in time to intubation between the two groups, it was calculated that a sample size of at least 149 participants in each group is necessary to detect a difference of 1 s in time to intubation in between the groups, if we allow a significance level of 5% and power our study at 85%. Thus, a total sample size to demonstrate the minimum effect size is 298, and assuming approximately 20% loss and rounding up to the nearest number, the total sample size required is 360 (180 in each group). The calculation was done using online sample size calculators http://powerandsamplesize.com and http://www.sample-size.netusing comparison of means for two-sided equality and the normal approximation using the *Z* statistics instead of the *T* statistics. The calculated sample size was concordant between the two calculators used.

We felt a 20% difference in time to intubate (TTI) as defined by us in the study would be clinically significant, as this difference means greater manipulations after the tube is inside the oral cavity resulting in more significant trauma and complications of intubation.

### Recruitment {15}

The patients posted for surgery, elective or emergency, under general anesthesia requiring endotracheal intubation will be assessed for eligibility criteria from the preoperative period. They will be recruited after they provide valid consent for the study. It is estimated that with the volume of surgeries and new trainees the institute sees every year, the sample size will be easy to achieve. Moreover, it is a routine procedure. Being a thesis topic, it was discussed in the departmental meeting and all 14 faculty and 31 residents were made aware of the protocol to make sure none of the eligible GA cases with ETT intubation by a trainee is missed.

### Assignment of interventions: allocation

#### Sequence generation {16a}

The enrolled participants will be randomized to one of two groups. A web-based random number generator (www.randomizer.org) will be used for generating codes under a simple randomization scheme. Participants will be randomized to either of the two groups at a 1:1 allocation ratio according to the random codes assigned by the web.

#### Concealment mechanism {16b}

The random codes will be written on a piece of paper and put in opaque brown envelopes which will be sealed. Once a patient has been enrolled, the operating room’s technical staff will open an opaque, sealed envelope containing the group allocation and mark the ETT accordingly. Allocation concealment will be ensured, as the randomization code will not be released until the patient has been recruited into the trial, which takes place after all baseline measurements have been completed in the pre-anesthesia checkup, the evening before surgery.

#### Implementation {16c}

Neither front-line care providers, investigators, nor participants will be aware of whether the next eligible participants will be receiving treatment or control intervention. One of the team members will enroll the participants and leave the set, sequentially numbered, generated by the research assistant, sealed opaque envelopes with the OT technician (OTT) responsible for anesthesia for the patient. The OTT will not be a part of the research team and will open the sealed envelopes to read the randomization sequence, which will be matched with the random number sequence generated earlier. He will be the one to mark the ETT at the designated site as per the number picked and the group it belongs to. The intubator will be asked to hold at the marked site after he/she confirms the optimal CL view. The assessors calculating time timings from the video will be blinded to the group the video belongs to.

### Assignment of interventions: blinding

#### Who will be blinded {17a}

The patient, the outcome assessor, and the data analyst will be blinded to the study intervention.

The intraoperative anesthesia team will not be blinded to the study.

#### Procedure for unblinding if needed {17b}

In infrequent circumstances which may hamper the patient’s safety, unblinding may be done.

### Data collection and management

#### Plans for assessment and collection of outcomes {18a}

All demographic and clinical data will be entered into paper-based case record forms.

Videographic data taken at the time of intubation will be stored with identifier codes. They will be analyzed separately by an experienced anesthesiologist from the investigating team who is not aware of the group allocations and a blinded assessor who is not part of the study team.

The case record forms can be collected by emailing the corresponding author of this manuscript.

#### Plans to promote participant retention and complete follow-up {18b}

As the patient will be with the same anesthesia team, retention will not be a significant concern once recruited. However, at the time of writing this manuscript for protocol submission (midway of the study), the COVID pandemic started—this affected the methodology of intubation—limiting the number of personnel in the room and the use of PPE, which might have affected the primary outcome; the number of intubations had also reduced, so we approached the IEC for allowing the inclusion of patients undergoing emergency surgeries ( non-COVID suspect) and those with any Mallampati scores ( previously we were excluding those with MPG III and IV) and for an interim analysis to assess for stopping the trial or amendment in sample size.

### Data management {19}

All data (case record forms and videos) will be stored in a password-protected computer in an Excel spreadsheet for further analysis. All measures will be taken to provide a backup of stored data in case of data loss.

### Confidentiality {27}

The participants’ identity and sensitive personal details will remain confidential with the principal investigators and will not be disclosed in any form.

### Plans for collection, laboratory evaluation, and storage of biological specimens for genetic or molecular analysis in this trial/future use {33}

No biological specimens will be collected in this study.

### Statistical methods

#### Statistical methods for primary and secondary outcomes {20a}

A researcher blinded to the group allocation will perform the statistical analysis of all randomized patients using the SPSS for Windows software (ver. 26.0; SPSS Inc., Chicago, IL, USA) on completion of the study.

Flowchart

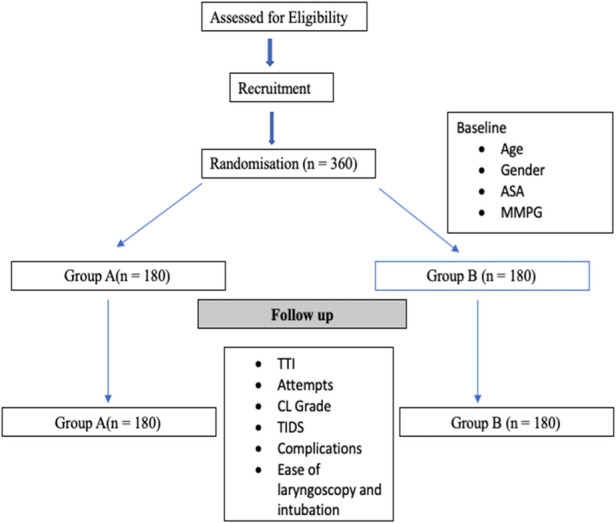



For presenting the demographic data, the categorical data (like gender, SA status, gender) will be presented as a percentage of the total, while the quantitative data will be presented as means or medians with standard deviations or interquartile range. The data will be tabulated with standardized differences reported between groups.

The primary outcome will be described in seconds. The descriptive statistics that will be used is a standardized difference of means or proportions accordingly. All the secondary outcomes data, namely time to intubation and the IDS, are quantitative and will first undergo a normality test either the Shapiro-Wilk test or the Kolmogorov-Smirnov test to check the normality of the quantitative variables’ distribution. After this, the Mann-Whitney *U* test or independent *t*-test will be used, depending on whether the data is parametric or non-parametric, including the primary outcome variables. Accordingly, the data will be represented as mean with standard deviation or median with interquartile range.

### Interim analyses {21b}

An interim analysis is planned after one hundred and twenty cases and approval granted from the ethics committee. The senior-most researcher (ST) will have access to the results of the interim analysis.

### Methods for additional analyses (e.g., subgroup analyses) {20b}

Subgroup analysis is not planned for the study. But in the course of the study if the investigators find something which can be used for subgroup analysis, then it will be notified to the IRC, IEC, Trial registry, and the journal too. An appropriate descriptive statistics will be used for the same.

### Methods in analysis to handle protocol non-adherence and any statistical methods to handle missing data {20c}

Sensitivity analysis will be done to handle the missing data. Missing data will be handled with multiple imputations. Per-protocol and ITT analyses will both be done.

### Plans to give access to the full protocol, participant-level data, and statistical code {31c}

Reasonable access to the protocol and other documents can be made by requesting the corresponding author.

### Oversight and monitoring

#### Composition of the coordinating center and trial steering committee {5d}

Two senior anesthesiologists from the investigating team will review the adherence to the trial protocol and overall conduct of the trial every 2 months throughout the study period.

The institute research committee also oversees the regular progress of academic dissertation-based research.

#### Composition of the data monitoring committee, its role, and reporting structure {21a}

Since the study does not involve any new intervention and endotracheal intubation is a regular procedure done in operating rooms under senior anesthesiologists’ guidance, the IEC did not call for a DMC appointment.

#### Adverse event reporting and harms {22}

Endotracheal intubation is a very safe procedure, and no new intervention will be investigated.

Hence, we do not expect any unexpected adverse events to happen during the study.

#### Frequency and plans for auditing trial conduct {23}

The trial is conducted as a part of the thesis. Therefore, intermittently, there will be meetings among the researchers to find out the progress of the study as well as any lapses or modifications required in the same.

#### Plans for communicating important protocol amendments to relevant parties (e.g., trial participants, ethical committees) {25}

This study was approved by the Institutional Ethics Committee of All India Institute of Medical Sciences, Bhubaneswar, on 15 July 2019 and registered at ctri.nic.in (CTRI/2019/09/021201) on 12 September 2019.

Any modifications in the protocol pertaining to eligibility criteria, outcomes, or analyses will be discussed among the researchers first following the audit. This will be informed and approved by the Departmental academic lead, Head of the department, Institute Research Committee, and Institutional Ethics Committee. After their approval, the modifications will be uploaded to the trial registration body (CTRI). The changes especially in the eligibility criteria will also be notified to other faculty and residents so that they can notify the researchers regarding the cases as per the new protocol.

## Dissemination plans {31a}

The results obtained from this study will be disseminated at anesthesia conferences (local and international meetings). The key findings will be reported in the trial registry. After completing the study, the report will be submitted for publication in an anesthesia journal, preferably an open-access journal.

## Discussion

It is already known that the first-pass intubation rate performed by trainees depends on their years of experience. Hence, systematic training of technical and non-technical skills is essential for procedural success. As per a review in the first 100 intubations done by a trainee, the early intubations’ major problems were esophageal intubations and achieving an optimal view.

Positioning of the patient and laryngoscopy technique are the two most important factors for achieving an optimal view during direct laryngoscopy. This study will first assess the optimal site of holding ET for successful LEI when performed by the trainees. The holding site can impact the visual axes and the torque applied to the endotracheal tube. Whether this will impact the incidence of complications like bleeding and sore throat is not known.

## Trial status

This study was approved by the Institutional Ethics Committee of AIIMS, Bhubaneswar, on 15 July 2019 and was registered in CTRI on 12 September 2019. The first participant was recruited on 16 September 2019, and currently, the study is in ongoing status**.**

## Data Availability

The data generated in this study can be shared after a reasonable request to the corresponding author of the manuscript. All investigators will be having access to the cleaned data sets. The Excel sheet and video recordings will be stored electronically. These will be password protected too.

## Abbreviations

ET: Endotracheal tube
ASA: American society of anesthesiologist
IDS: Intubation difficulty score
MPG: Mallampati grading
OT: Operating theater
SD: Standard deviation
